# Inhaled Corticosteroids and the Risk of Lung Cancer in Chronic Obstructive Pulmonary Disease Patients: A Systematic Review and Meta-Analysis

**DOI:** 10.1155/2022/9799858

**Published:** 2022-08-21

**Authors:** Amare Abera Tareke, Wondwosen Debebe, Addis Alem, Nebiyou Simegnew Bayileyegn, Taddese Alemu Zerfu, Andualem Mossie Ayana

**Affiliations:** ^1^Department of Biomedical Sciences, College of Medicine and Health Sciences, Wollo University, Dessie, Ethiopia; ^2^Department of Surgery, Faculty of Medicine, Jimma University, Jimma, Ethiopia; ^3^College of Medicine and Health Sciences, Dilla University, Dilla, Ethiopia; ^4^Global Academy of Agriculture & Food Security (GAAFS), University of Edinburg, UK; ^5^Department of Biomedical Sciences, Faculty of Medicine, Jimma University, Jimma, Ethiopia

## Abstract

**Background:**

The global prevalence of chronic obstructive pulmonary disease (COPD) is increasing, and the risk of lung cancer in these patients is high. The use of inhaled corticosteroids (ICSs) in COPD patients could help to decrease potential lung cancer risk. We planned to conduct this systematic review and meta-analysis to determine the role of ICS in the risk of lung cancer among COPD patients.

**Methods:**

A comprehensive search of PubMed, Science Direct, Google Scholar, and Cochrane library and a manual search of the list of references were conducted. Studies with cohort, case-control, and randomized clinical trial designs for any ICS use reporting the incidence/hazard ratio (HR) of lung cancer were included. The random-effects model was used to pool hazard ratios. Subgroup analysis and metaregression analysis were employed. Funnel plot and Egger regression test were used to assess publication bias.

**Results:**

Combining the results of 14 observations, the pooled HR for cancer risk reduction was 0.69 (95% CI 0.59-0.79), *p* value ≤ 0.001. The use of ICS in COPD patients showed a 31% reduction in the risk of lung cancer. Subgroup meta-analysis showed a significant reduction in the risk of lung cancer as well.

**Conclusion:**

The use of ICS in COPD patients reduces the risk of lung cancer. The risk reduction was independent of smoking status and latency period. Future studies should focus on the optimum dose and controlling confounders like asthma.

## 1. Introduction

With uneven distribution around the world, the prevalence of chronic obstructive pulmonary disease (COPD) is increasing [1]. Over a 44% increment in the prevalence of COPD was observed from 1990 to 2015, and 174.5 million individuals suffered from COPD. In 2015, 3.2 million people died worldwide which is an 11.6% increment compared to 1990 [2]. Mortality has been an important outcome in COPD, and it has become one of the leading causes of death in the world. Causes of mortality in COPD vary depending on the population; a randomized clinical trial of smoking cessation and inhaled bronchodilator therapy indicated cancer was the most common cause of mortality (33%) [3]. However, in a pooled analysis of seven randomized clinical trials among patients with stable COPD, the leading cause of death was respiratory disease [4].

A consistent positive association between COPD and lung cancer is common. The annual incidence of lung cancer was 2 to 4-fold higher in patients with prior COPD [5, 6]. From 21 studies conducted between 1997 and 2018, the pooled prevalence of lung cancer in COPD patients was 2.79%, and people with COPD are 6.35 times more likely to develop lung cancer than controls [7]. Smoking has been associated with an increased risk of lung cancer in COPD patients [8] and advocated as the link. Recent studies outline the risk of lung cancer remains higher even without smoking [9]. In a large Korean national cohort study, COPD was a strong independent risk factor for lung cancer incidence in never smokers, indicating these patients are at high risk of lung cancer irrespective of smoking status [10]. Airway remodeling, epithelial-mesenchymal transition, and to the lesser extent infections that predispose to airway inflammation and epithelial activation and further exacerbate oxidative injury are involved in the pathogenesis of COPD [11]. Epithelial-mesenchymal transition, by altering the cellular architecture of the airway, plays an important role in disease progression, development of cancer, and tumor metastasis, which increases cancer-related mortality [12].

Mortality from lung cancer is higher in inflammatory lung conditions. Forced expiratory volume in the first second (FEV1) and C-reactive protein (CRP) significantly predict mortality from lung cancer with hazard ratio (HR) = 2.13 for FEV1 < 90% and HR = 3.38 for CRP > 2 mg/dl [13]. Chronic inflammation induces immune components including interleukin 1*β* (IL-1*β*), IL-10, IL-17A, cyclooxygenase 2 (COX 2), and T-helper 1 (Th 1) cells. The resultant effects impair cytotoxic T cell effector function, activate macrophages, and degranulate neutrophils [14, 15]. These inflammatory molecules cause genomic instability, suppress tumor immune surveillance, and tumor-promoting inflammation, which contribute to proliferation including tumor growth and metastasis [14].

Pharmacologic treatments for COPD include decreasing airway smooth muscle contraction, airway inflammation, mucus production, respiratory infection, and replacement of alpha-1 antitrypsin deficiency. Inflammatory conditions in COPD can be managed with corticosteroids, phosphodiesterase inhibitors, and long-term macrolides [16]. In the recent (2020) Global Initiative for Chronic Obstructive Pulmonary Disease (GOLD) report, there was no conclusive evidence indicating modification in the decline of FEV1 or mortality with regular use of inhaled corticosteroids (ICSs) in COPD [17]. However, in moderate COPD, a slower decline in FEV1 and a reduction in the occurrence of exacerbation were reported [17, 18]. Nevertheless, GOLD recommends the use of ICS in combination with bronchodilators for COPD exacerbations, and the use of ICS for COPD patients is common in clinical practice.

ICS use for COPD has mixed outcomes; deleterious effects like pneumonia [19] and tuberculosis [20] are implied. On the other hand, the ICS prescription for COPD modulates inflammation. Treatment with fluticasone propionate for 10 weeks alleviated increment in markers of inflammation and adhesion molecules and boosted antioxidant capacity [21]. A double-blind placebo-controlled clinical trial also provides evidence of ICS suppression of systemic inflammation. Withdrawal of inhaled corticosteroids increased serum CRP level and reintroduction of inhaled fluticasone suppressed CRP level [22]. These conflicting pieces of evidence create uncertainty in the use of ICS in COPD and subsequent benefits to these specific individuals.

As the global prevalence of COPD is increasing and the risk of lung cancer in these patients is high; the use of ICS in COPD patients could help to decrease potential lung cancer risk. A systematic review by Raymakers et al. [23] in 2016 on ICS use and incidence/mortality in lung cancer appraises two separate results from clinical trials and observational studies. Although they opted not to pool due to methodological reasons, randomized controlled trials (RCTs) indicated no benefit and observational studies pointed out the possibility of beneficial effects. Recent meta-analyses on the benefits of ICS in the incidence of lung cancer pointed out the significant role of these drugs in risk reduction [24, 25]. We conducted this systematic review and meta-analysis to determine the role of ICS on the risk of lung cancer in COPD patients. This study added evidence to the current knowledge with a robust search and additional literature.

## 2. Methods

### 2.1. Eligibility Criteria

Our inclusion criteria were as follows: (1) studies with a cohort, case-control, and randomized clinical trial designs for any inhaled corticosteroids including beclomethasone, budesonide, triamcinolone, fluticasone, ciclesonide, mometasone, flunisolide; (2) the follow-up period should be at least 12 months; (3) study participants with COPD; (4) the intervention should be ICS, the control group with placebo or other drugs with a specified name; and (5) data on the incidence of lung cancer including zero events in the course of follow-up.

In vitro and animal studies, review articles, incomplete articles, conference proceedings, and duplicates were excluded. Due to feasibility, articles published in the English language/have English version were included. All the abovementioned designed research conducted on adults up to November 2021 were included.

### 2.2. Search Strategy

PubMed, Science Direct, Google Scholar, and Cochrane library searched for the following terms without publication year restrictions: COPD, “obstructive pulmonary disease,” “Pulmonary Disease, Chronic Obstructive,” “inhaled corticosteroids,” Beclomethasone, budesonide, triamcinolone, fluticasone, mometasone, ciclesonide, flunisolide, lung neoplasms, lung cancer, non-small lung carcinoma, small lung carcinoma, adenocarcinoma, “carcinoma, squamous cell” search words used alone or in combination using Boolean operators. Unpublished articles were searched from clinical trial registration platforms. Preprint articles were also retrieved from websites. A manual search was conducted by screening the reference lists of included studies. Reports were compiled according to the Preferred Reporting Items for Systematic Reviews and Meta-Analysis (PRISMA) guideline [26].

### 2.3. Study Selection

Two reviewers independently scanned all titles and abstracts that indicated the use of corticosteroids by a patient with COPD and the risk of lung cancer. After obtaining full reports of potentially relevant studies, the same reviewers independently assessed the full-text articles. Disagreements regarding eligibility were solved by the third reviewer through consensus.

### 2.4. Data Extraction and Outcome Measurement

General study details including author, publication year, study design, country, follow-up period and duration, age of participants, the sample size in both groups, types of treatment, latency period, adjusted variables, and hazard ratio were extracted from each study with predetermined data abstraction format. The outcome of interest was the risk of lung cancer, incidence, or HR. In studies that had more than two groups and reported more than one hazard ratio, the HRs will be taken accordingly. In this case, the sample size of the control group was divided by the number of groups/experimental arms to avoid double counting.

### 2.5. Risk of Bias Assessment

The methodological quality of observational studies was assessed using the Newcastle Ottawa Scale (NOS) [27]. The risk of bias for included RCT was judged per the Cochrane Collaboration Risk of Bias Tool [28], for reporting of sequence generation, allocation concealment, use of blinding of participants and personnel, loss to follow-up, and other biases.

### 2.6. Data Analysis

We performed a meta-analysis to pool HR with a 95% confidence interval (CI) from dichotomous data using random effects, inverse variance method. Statistical heterogeneity was measured by *I*^2^ static, and we consider percentages of around *I*^2^ = 25%, *I*^2^ = 50%, and *I*^2^ = 75% as low, medium, and high heterogeneity, respectively [29].

Subgroup meta-analysis was performed for potential sources of clinical and methodological heterogeneity. Studies subgrouped based on the status of adjustment for smoking, duration of follow-up period, consideration of time bias, and so on. Meta-regression analysis was performed for potential covariates. Potential publication bias was assessed using a funnel plot and Egger's regression test. *p* value ≤ 0.05 cut-point was used to declare statistical significance. To detect the robustness of the results, a sensitivity analysis was conducted by sequential elimination of each study from the pool. All analyses were performed using the STATA software (Version 14, StataCorp, Texas, USA).

## 3. Results

### 3.1. Literature Search Result

**Figure 1:**
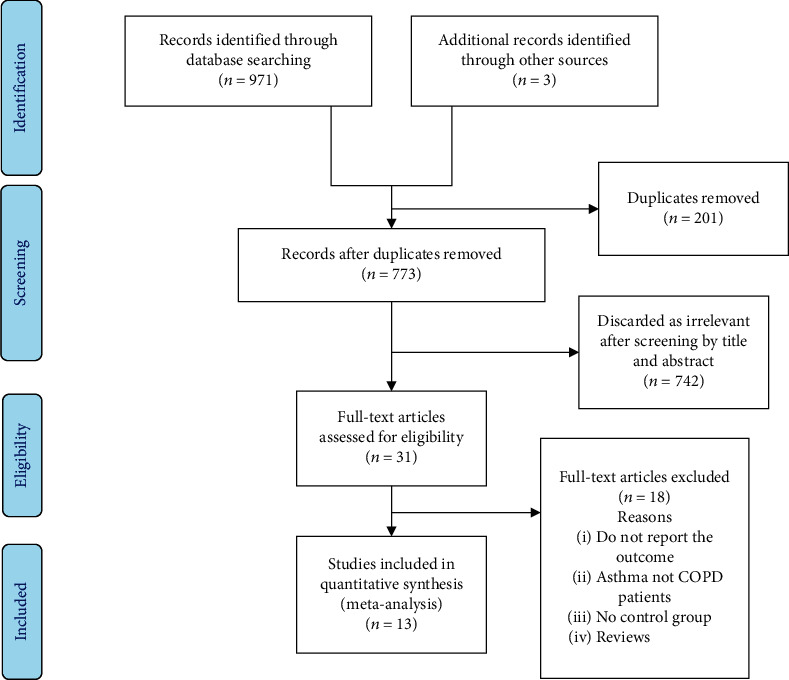
PRISMA flow diagram of study selection to estimate HR for risk of lung cancer among COPD patients taking ICS and controls.

A total of 971 potentially eligible studies identified from the four databases were searched, and three additional articles were retrieved from other sources. Two hundred one duplicates were removed with 773 studies left to deal. After screening the titles and abstracts of these studies, 742 papers discarded as irrelevant. Thirty-one studies selected for full content screening, and 13 articles met the inclusion criteria. The detail of the literature selection is given in Figure 1, with the PRISMA flow diagram.

### 3.2. Characteristics of Original Studies

Nine cohort studies, three case-control, and one randomized controlled trial, a total of 13 studies, (with 14 observations), were included. The mean follow-up time ranges from 3 to 6.8 years, and publication year ranges from 1999 to 2019. Parimon et al. [30] reported two hazard ratios for ICS dose less than and greater than 1200 *μ*g/day without shared HR. We took these two HRs as two observations, and the total observations are 14 with 13 unique studies.  Table 1 shows the characteristics of included articles including treatment regimen and confounder adjustments.

### 3.3. Meta-Analysis

**Figure 2:**
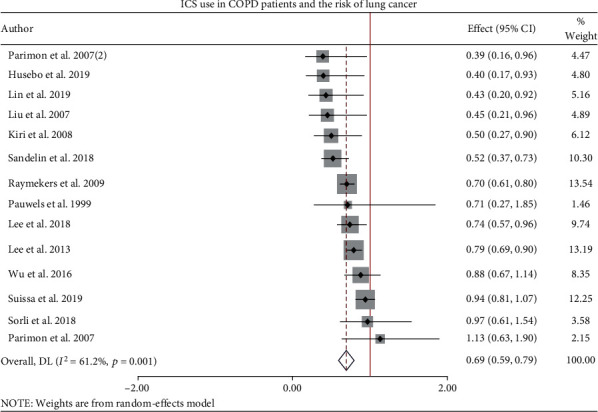
Forest plot of pooled HR of ICS use in COPD patients and the risk of lung cancer.

Given in the forest plot (Figure 2), pooling the results of 14 reports, the pooled HR for cancer risk reduction was 0.69 (95% CI 0.59-0.79), *p* value ≤ 0.001. The use of ICS in COPD patients showed a 31% reduction in the risk of lung cancer with a mean follow-up time of 3-6.8 years. However, there is borderline moderate heterogeneity *I*^2^ = 61.2%. Subgroup meta-analysis was conducted on relevant factors including study design, continent, smoking status, and latency period. All factors fail to change the significant reduction in the risk of lung cancer among COPD patients taking ICS (Table 2). We fail to pool HRs for RCTs because a single study was eligible for this analysis, and the effect was not significant.

**Figure 3:**
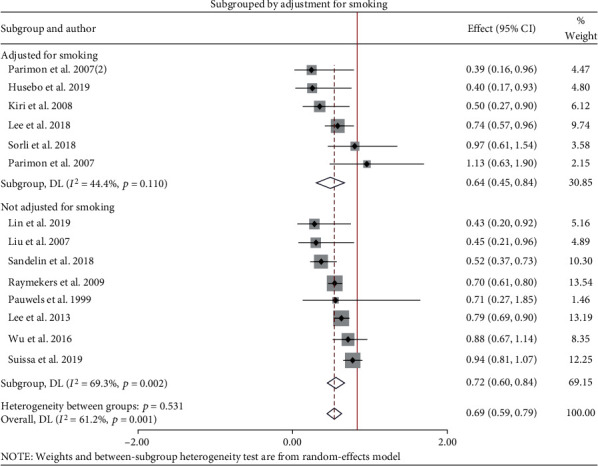
HR of the risk of lung cancer subgrouped by adjustment for smoking status of patients.

Some studies had data for smoking and adjusted it for analysis, while others have no data. We dichotomize smoking status adjusted (if adjusted in analysis or all participants were nonsmokers) and not adjusted (if the study lacks data or unadjusted during analysis). In both cases, there is a significant risk reduction, and adjustment for smoking resulted in a greater reduction of 36% (vs. 28%). Subgroup analysis based on adjustment for smoking is given in Figure 3.

Cohort studies reported greater significant reduction in risk, than case control studies, 32% and 26%, respectively, with moderate heterogeneity in cohort studies. A single RCT included in this analysis insignificant result. Continent wise, researches conducted in Europe had pronounced risk reduction than elsewhere. There was also significant risk reduction in Asia and America (USA and Canada) with 28% and 23%, respectively, although lower than the European studies.

The latency period is the period between the entrance to follow-up and the development of lung cancer. A one-year latency period was allowed in some studies, and we performed a subgroup analysis to test if the latency period had affected the outcome. As we can see in Table 2, there is a significance risk reduction in both cases. Allowance of at least 1-year latency period had lower reduction with HR = 0.78 (95% CI 0.63-0.94), *p* value ≤ 0.001. Without latency period, risk reduction was higher (HR = 0.61 (95% CI 0.47-0.76), *p* value ≤ 0.001). Forest plots for subgroup meta-analysis based on study deign, continent of the study, and latency period are shown in the supplementary material, supplementary figures 1A-C, respectively.

We also conducted a metaregression analysis to identify covariates affecting the outcome. Metaregression was undertaken on study design, continent, smoking adjustment, and latency period; the result showed none of them were significant covariates. Metaregression analysis results are given in Table 3.

### 3.4. Publication Bias

**Figure 4:**
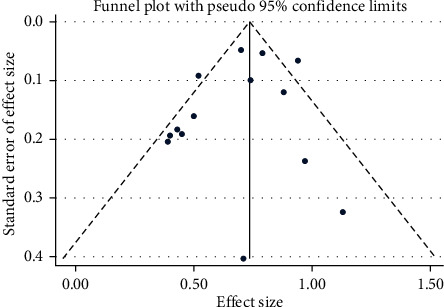
Funnel plot for detection of publication bias.

The visual inspection of the funnel plot, Figure 4, shows no asymmetry, and Egger's regression test was insignificant (*p* value = 0.476). Sensitivity analysis was performed by sequential elimination of each study from the pool, and there was single study affecting the finding, indicated in the supplementary material (Supplementary figure 2 and table S2).

## 4. Discussion

This systematic review and meta-analysis pooled the risk of lung cancer in COPD patients with and without ICS use. We found that ICS use in COPD patients confers up to 31% (95% CI 21%-41%) significant risk reduction. The evidence was synthesized from observational and interventional studies (cohort, case-control, and RCTs). Although it is well established that COPD is an independent risk factor for lung cancer, the mechanism by which ICS use could reduce the risk is not well understood and more studies are being conducted.

Chronic inflammation is in the first line of proposed mechanisms. Thomsen et al. [31] measured inflammatory markers like CRP, fibrinogen, and leukocyte count. When markers were elevated, the HR for lung cancer was increased fourfold, HR = 4.00 (95% CI 2.12-7.54). Chronic inflammation increases emphysema-like alveolar space enlargement promoting smoke carcinogen-induced tumorigenesis [32]. The other proposed mechanism is the alteration in redox balance. Greater levels of radical oxygen species production beyond the normal scavenging capacity of the body's antioxidant system cause peroxidation of membrane phospholipids, modification of nuclear DNA, and alteration of proteins [15]. ICS for COPD decreased inflammatory responses [33, 34], though there are still contrary findings [35] regarding the alleviation of inflammation.

The capability of inflammatory cells to produce arachidonic acid metabolites was decreased after fluticasone inhalational treatment [36]; inhalational budesonide for 6 months reduced the percentage of neutrophils and IL-8 concentration in Broncho-alveolar lavage [37]. A meta-analysis conducted on the immune-regulatory function of ICS [38] provided evidence of reduced neutrophil and lymphocyte count and a significant increase in macrophages. This explains the beneficial effects on exacerbation and increasing the risk of pneumonia in these patients. More importantly, the risk of lung cancer could be reduced along with the reduction of chronic inflammation and redox imbalance.

This meta-analysis showed ICS treatment has reduced the risk of lung cancer. The risk reduction was independent of study design (more clinical trials should be conducted to estimate the risk in RCTs), smoking adjustment, or latency period. In observational studies, both cohort and case-control, a significant risk reduction was observed. However, the results of RCTs were not pooled because of insufficient eligible studies. Adjustment for smoking still provided significant risk reduction, but in comparison with unadjusted risk, the risk reduction was greater.

Incorporating a latency period in the analysis of primary studies was an important component of reducing time-related bias. An incident case that occurred within days of the entrance to follow-up will not indicate the effect of treatment. Since lung cancer is diagnosed at the advanced stage, it is likely to have been present prior to the clinical diagnosis. This has been the source of variation in different reports. Given the biology of tumor growth in lung cancer, a year latency period was applied in some studies [39]. Subgroup analysis conducted with latency period also indicated the importance of this variable. Although it did not affect the significance of ICS, the magnitude of reduction was different (22% for studies with a latency period versus 39% lung cancer diagnosis without latency period). This difference is high, and it is important to consider latency period in future investigations.

Although this study provided comprehensive evidence from good quality literature (quality of studies indicated tables S1A-C), the findings of this study should be interpreted curiously. First, because of inconsistent reporting and unable to pool it, the dose of ICS was not analyzed. The dose of ICS is an important factor in the survival or risk of other comorbidities among COPD patients [30, 40]. Second, the diagnosis of COPD in the included studies is worth mentioning. Asthma might have played an important confounding factor that affects several observational studies of ICS effectiveness [41]. Among patients recruited for the follow-up, some might have asthma, and others might have asthma-COPD overlap. Patients with asthma are more likely to receive ICS and less likely to develop lung cancer. The residual confounding of asthma cannot be ruled out. Besides asthma, other possible risk factors [42] of lung cancer including interstitial lung disease were not evaluated and adjusted in most of the original literature; the presence of such comorbidities is likely to affect the development of lung cancer.

## 5. Conclusion

The use of ICS in COPD patients reduced the risk of lung cancer. The risk reduction was independent of smoking status and latency period. Future studies should focus on the optimum dose and controlling confounders like asthma.

## Figures and Tables

**Table 1:** Description of included studies in ICS use and the risk of lung cancer in COPD patients.

Author-year	Country	Design/study period	Mean follow-up in years ± SD	Mean age ± SD	Sample size	Treatment	Latency period (years)	Adjusted for
Parimon et al. 2007 [30]	USA	Cohort 1996-2001	3.84 (2.08-4.29)	66 ± 10^t^ 64 ± 11^c^	517^t^ 9957^c^	Triamcinolone, beclomethasone, flunisolide, fluticasone	1	Age, smoking status, smoking intensity, history of non-lung-cancer malignancy, coexisting illness, bronchodilator use
Raymekers et al. 2009 [43]	Canada	Cohort 1997-2007	5.2 ± 2.3	70.7 ± 11	28314^t^ 11312^c^	ICS	1	Age, sex, neighborhood income quartile, regional health service, prescriptions filled, hospitals encountered, inpatient hospital stays, physician encounters, comorbidity
Suissa et al. 2019 [44]	Canada	Cohort 2000-2014	4.9	71 ± 10	58177	Triamcinolone, beclomethasone, flunisolide, fluticasone, budesonide, mometasone, ciclesonide	1	Age, sex, COPD hospitalizations and exacerbations in the prior year, comorbidity including CVD, DM, renal diseases, other cancers, dementia, rheumatic diseases
Lee et al. 2013 [45]	Korea	Case-control 2007-2010	3	68.7^t^ 68.1^c^	9177^t^ 37048^c^	Triamcinolone, beclomethasone, flunisolide, fluticasone, budesonide, ciclesonide	NR	Other medications, overall comorbidity, cumulative dose
Lee et al. 2018 [46]	Korea	Case-control 2002-2013	4 ± 2.48	68	265^t^ 1060^c^	Triamcinolone, beclomethasone, flunisolide, fluticasone, budesonide, ciclesonide	1	Smoking intensity, BMI, comorbidity, household income
Kiri et al. 2008 [47]	UK	Case-control 1989-2003	NR	70.7 ± 6.9^t^ 70.8 ± 6.6^c^	124^t^ 1470^c^	ICS^E^ LABD^C^	NR	Use of other respiratory medications, comorbidity including asthma, duration of smoking, time from smoking cessation
Liu et al. 2007 [48]	Taiwan	Cohort 1997-2009	10.2		1290^t^ 12396^c^	Fluticasone, budesonide	NR	Age, income, comorbidity
Pauwels et al. 1999 [49]	9 European countries	RCT	NR	52.5 ± 7.5^t^ 52.4 ± 7.7^c^	634^t^ 643^c^	Budesonide	NR	Unreported confounders
Sorli et al. 2018 [50]	Norway	Cohort 1996-2008	NR	61^t^ 58^c^	1095^t^ 1946^c^	Beclomethasone, fluticasone, budesonide	6	Sex, smoking pack-years
Sandelin et al. 2018 [51]	Sweden	Cohort 1999-2009	NR	68.9 ± 8.5^t^ 68.0 ± 11.4^c^	594^t^ 19300^c^	ICS	NR	Age at COPD diagnosis, gender, asthma, educational level, marital status, income prior to index, medication, comorbidities
Wu et al. 2016 [52]	Taiwan	Cohort 2003-2010	NR	65.0^t^ 65.0^c^	8812^t^ 35,252^c^	Beclomethasone, budesonide, fluticasone, and ciclesonide	NR	Sex, age, medications, comorbidities, inpatient and outpatient visits for respiratory diseases, and urbanization
Lin et al. 2019 [53]	Taiwan	Cohort 2000-2010	NR		2,682t 2,682^c^	ICS	NR	Gender, age, work occupation, and comorbidities
Husebo et al. 2019 [54]	Norway	Cohort 2009-2010	NR	63.5^t^ 63.5^c^	64t 58^c^	ICS	9	Age, sex, smoking status, pack-years smoked, and body composition

NR: not reported. ^t^ICS-treated group. ^c^Control (non-ICS group). RCT: randomized controlled trial; CVD: cardiovascular disease; BMI: body mass index; DM: diabetes mellitus; COPD: chronic obstructive pulmonary disease; ICS: inhaled corticosteroid; SD: standard deviation.

**Table 2:** Subgroup analysis of the pooled HR of ICS use in COPD patients and the risk of lung cancer.

Subgroup	Number of studies	HR (95% CI)	*Z* (*p* value)	*I* ^2^ (*p* value)
Total	14	0.69 (0.59-0.79)	13.56 (<0.001)	62.1% (0.001)
Design				
Cohort	10	0.68 (0.54-0.82)	9.31 (<0.001)	70.2 (0.<001)
Case control	3	0.74 (0.61-0.86)	11.36 (<0.001)	32.6% (0.23)
RCT	1	0.71 (-0.08-1.50)	1.76 (0.078)	- -
Continent				
Europe	4	0.55 (0.41-0.69)	7.67 (<0.001)	2.2% (0.39)
America	5	0.77 (0.55-0.99)	6.97 (<0.001)	77.5% (<0.001)
Asia	5	0.72 (0.59-0.86)	10.70 (<0.001)	44.5% (0.13)
Smoking				
Adjusted	6	0.64 (0.45-0.84)	6.38 (<0.001)	44.4% (0.11)
Not adjusted	8	0.72 (0.60-0.84)	11.76 (<0.001)	69.3% (<0.001)
Latency period				
Yes	6	0.78 (0.63-0.94)	10.12 (<0.001)	64.6% (0.01)
No	8	0.61 (0.47-0.76)	8.25 (<0.001)	57.96% (0.02)

**Table 3:** Metaregression analysis of possible covariates for lung cancer risk.

	Variable	Coefficients	*p* value
1	Study design	0.100	0.358
2	Continents	-0.047	0.575
3	Smoking status	0.180	0.051
4	Latency period	-0.27	0.213

## Data Availability

All data generated or analyzed during this study are included in this published article/as supplementary information files.

## Abbreviations

CI: Confidence interval
COPD: Chronic obstructive pulmonary disease
COX 2: Cyclooxygenase 2
CRP: C-reactive protein
FEV1: Forced expiratory volume in the first second
GOLD: Global Initiative for Chronic Obstructive Pulmonary Disease
HR: Hazard ratio
ICSs: Inhaled corticosteroids
NOS: Newcastle Ottawa Scale
PRISMA: Preferred Reporting Items for Systematic Reviews and Meta-Analysis
RCTs: Randomized controlled trials.

## Supplementary Materials

Supplementary Materials Table S1A: methodological quality of cohort studies assessed using the Newcastle Ottawa Scale. Table S1B: methodological quality of case control studies assessed using the Newcastle Ottawa Scale. Table S1C: risk of bias for the randomized controlled trial. Table S2: sensitivity analysis by sequential elimination of each study from the pool. Supplementary Figure 1A: subgroup analysis of lung cancer risk in ICS use among COPD patients (by design). Supplementary figure 1B: subgroup analysis of lung cancer risk in ICS use among COPD patients (by continent). Supplementary figure 1C: subgroup analysis of lung cancer risk in ICS use among COPD patients (subgrouped by latency period). Supplementary Figure 2: sensitivity analysis for visual inspection.
