# Supplementary material for: Inhaled Corticosteroids and the Risk of Lung Cancer in Chronic Obstructive Pulmonary Disease Patients: A Systematic Review and Meta-Analysis
Source: Pulm Med. 2022 Aug 21;2022:9799858. doi: 10.1155/2022/9799858 (PMC9420625; doi:10.1155/2022/9799858)
Supplement: Supplementary Materials — Table S1A: methodological quality of cohort studies assessed using the Newcastle Ottawa Scale. Table S1B: methodological quality of case control studies assessed using the Newcastle Ottawa Scale. Table S1C: risk of bias for the randomized controlled trial. Table S2: sensitivity analysis by sequential elimination of each study from the pool. Supplementary Figure 1A: subgroup analysis of lung cancer risk in ICS use among COPD patients (by design). Supplementary figure 1B: subgroup analysis of lung cancer risk in ICS use among COPD patients (by continent). Supplementary figure 1C: subgroup analysis of lung cancer risk in ICS use among COPD patients (subgrouped by latency period). Supplementary Figure 2: sensitivity analysis for visual inspection. [file 9799858.f1.docx]

Supplementary materials

Risk of bias for included studies

Table S1A methodological quality of cohort studies assessed using the Newcastle Ottawa Scale

| Author - year | Selection | | | | Comparability | | Outcome | | | Total |
| --- | --- | --- | --- | --- | --- | --- | --- | --- | --- | --- |
|  | a | b | c | d | e | f | g | h | 1 |  |
| Primon et al. 2007 | 1 | 1 | 1 | 1 | 1 | 1 | 1 | 1 | 1 | 9 |
| Raymekers et al. 2009 | 1 | 1 | 1 | 1 | 0 | 1 | 1 | 1 | 1 | 8 |
| Suissa et al. 2019 | 1 | 1 | 1 | 1 | 0 | 1 | 1 | 1 | 1 | 8 |
| Sorli et al. 2018 | 1 | 1 | 0 | 1 | 1 | 0 | 1 | 1 |  | 7 |
| Sandelin et al. 2018 | 1 | 1 | 1 | 1 | 0 | 0 | 0 | 1 | 1 | 6 |
| Liu et al. 2018 | 1 | 1 | 1 | 1 | 0 | 1 | 0 | 1 | 1 | 7 |
| Wu et al. 2016 | 1 | 1 | 1 | 1 | 0 | 1 | 0 | 1 | 1 | 7 |
| Lin et al. 2019 | 1 | 1 | 1 | 1 | 0 | 1 | 0 | 1 | 1 | 7 |
| Husebø et al. 2019 | 1 | 1 | 1 | 1 | 1 | 0 | 1 | 1 | 1 | 8 |

a. Representativeness of the exposed cohort

b. Selection of the non-exposed cohort

c. Ascertainment of exposure

d. Demonstration that outcome of interest was not present at start of study

e. Comparability of cohorts on the basis of the design or analysis (adjusted for smoking)

f. Comparability of cohorts on the basis of the design or analysis (adjusted for comorbidities)

g. Assessment of outcome

h. Was follow-up long enough for outcomes to occur

i. Adequacy of follow-up of cohort

Table S1B methodological quality of case control studies assessed using the Newcastle Ottawa Scale

| Author - year | Selection | | | | Comparability | | Exposure | | | Total |
| --- | --- | --- | --- | --- | --- | --- | --- | --- | --- | --- |
|  | a | b | c | d | e | f | g | h | i |  |
| Lee et al. 2013 | 1 | 1 | 1 | 1 | 0 | 1 | 0 | 1 | 1 | 7 |
| Lee et al. 2018 | 1 | 1 | 1 | 1 | 1 | 1 | 1 | 1 | 1 | 9 |
| Kiri et al. 2008 | 1 | 1 | 1 | 0 | 1 | 0 | 1 | 1 | 1 | 7 |

Table S1C risk of bias for the randomized controlled trial

|  | Bias | Pauwels et al. 1999 |
| --- | --- | --- |
|  | Random sequence generation | Unclear risk |
|  | Allocation concealment | Low risk |
|  | Blinding of participants and personnel | Low risk |
|  | Blinding of outcome assessment | Low risk |
|  | Incomplete outcome data | High risk |
|  | Other bias | Low risk |


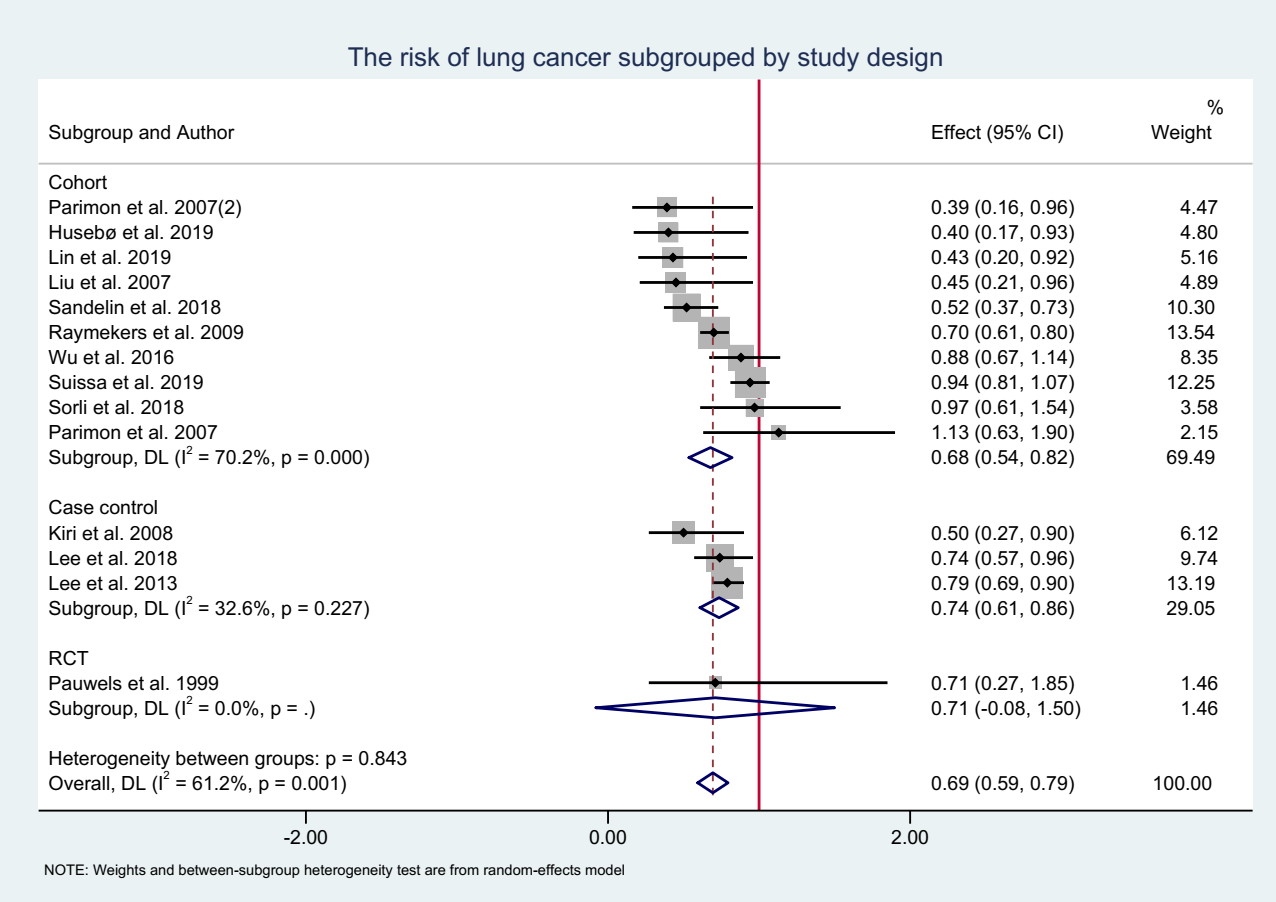
Supplementary figure 1A: subgroup analysis of lung cancer risk in ICS use among COPD patients (By design).


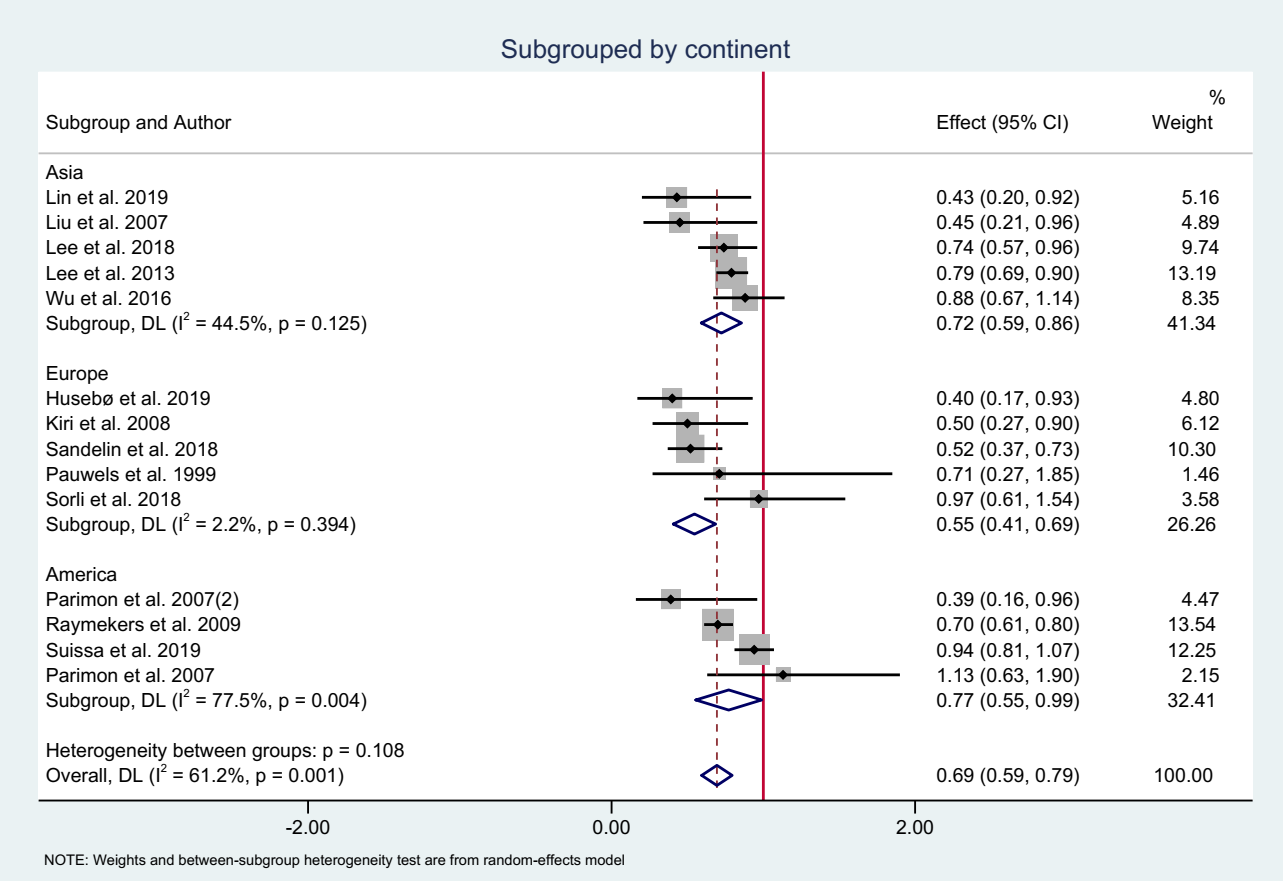


Supplementary figure 1B: subgroup analysis of lung cancer risk in ICS use among COPD patients (By continent)


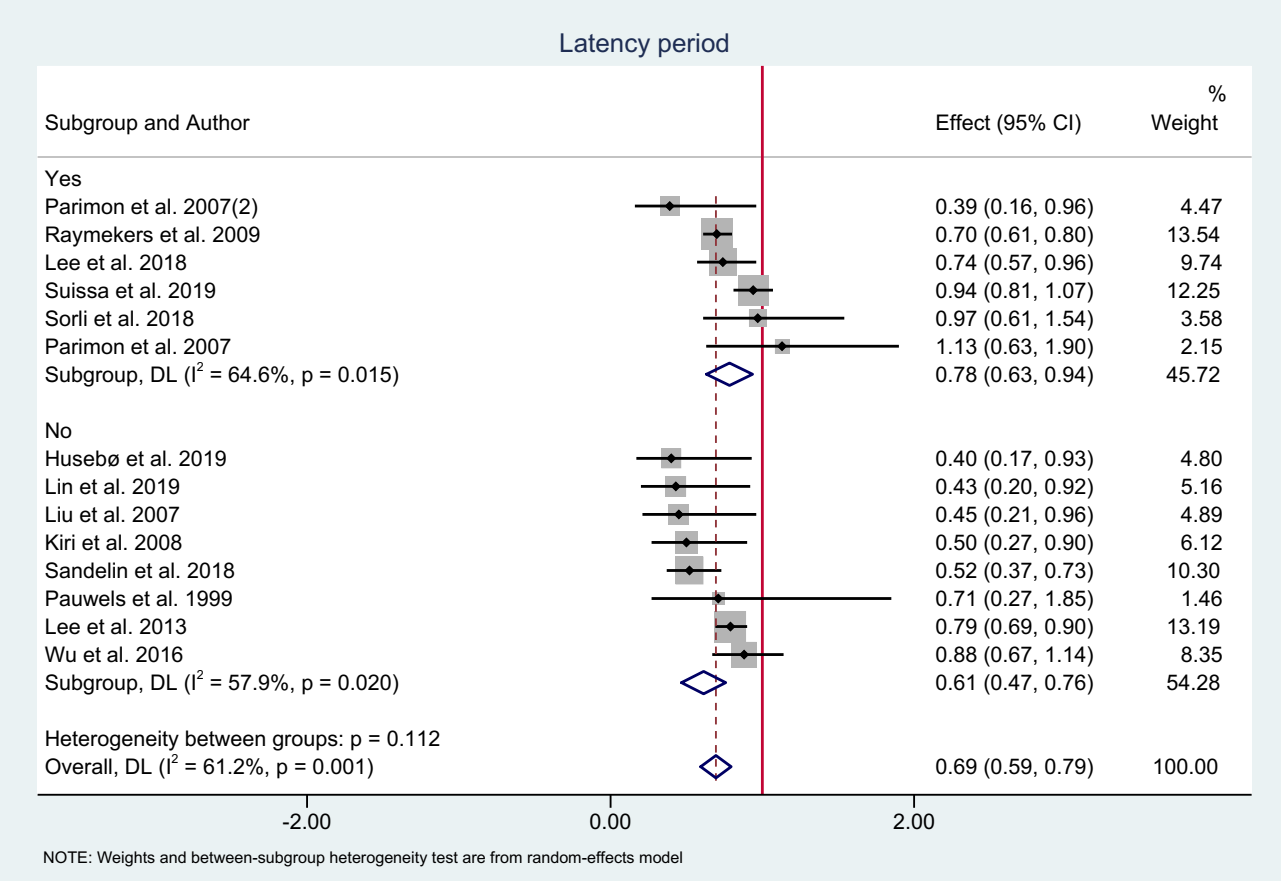


Supplementary figure 1C: subgroup analysis of lung cancer risk in ICS use among COPD patients (sub grouped by latency period)

Table S2: Sensitivity analysis by sequential elimination of each study from the pool

| Study omitted | Estimate | Lower CI(95%) | Upper CI(95%) |
| --- | --- | --- | --- |
| Parimon et al. 2007 | 0.68787807 | 0.58758414 | 0.78817207 |
| Parimon et al. 2007(2) | 0.71286869 | 0.61338401 | 0.81235331 |
| Raymekers et al. 2009 | 0.69080704 | 0.57193482 | 0.80967927 |
| Suissa et al. 2019 | 0.66875803 | 0.57487601 | 0.76264 |
| Lee et al. 2013 | 0.67955798 | 0.56332493 | 0.79579097 |
| Lee et al. 2018 | 0.69009477 | 0.58063644 | 0.7995531 |
| Kiri et al. 2008 | 0.70987982 | 0.60777861 | 0.81198102 |
| Liu et al. 2007 | 0.7098977 | 0.60888487 | 0.81091052 |
| Pauwels et al. 1999 | 0.69197112 | 0.58908451 | 0.79485774 |
| Sorli et al. 2018 | 0.68664402 | 0.58461815 | 0.78866988 |
| Sandelin et al. 2018 | 0.71988648 | 0.61956936 | 0.8202036 |
| Wu et al. 2016 | 0.67986131 | 0.57449752 | 0.78522509 |
| Lin et al. 2019 | 0.71202672 | 0.61168033 | 0.8123731 |
| Husebø et al. 2019 | 0.71260059 | 0.61289257 | 0.81230861 |
| Combined | 0.69696005 | 0.59729591 | 0.7966242 |


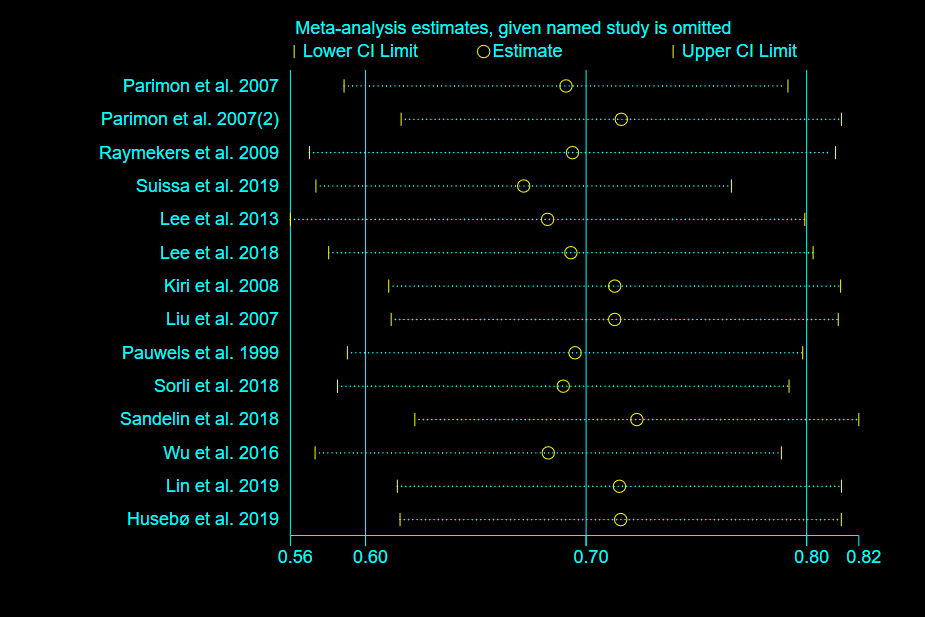


Supplementary figure 2: sensitivity analysis for visual inspection
